# Initiating decision-making conversations in palliative care: an ethnographic discourse analysis

**DOI:** 10.1186/1472-684X-13-63

**Published:** 2014-12-23

**Authors:** Emmanuelle Bélanger, Charo Rodríguez, Danielle Groleau, France Légaré, Mary Ellen Macdonald, Robert Marchand

**Affiliations:** Department of Social and Preventive Medicine, Université de Montréal, 7101 Parc Avenue, Montreal, H3N 1X9 Quebec Canada; Department of Family Medicine, McGill University, Montreal, Canada; Department of Psychiatry, McGill University, Montreal, Canada; Department of Emergency and Family Medicine, Université Laval, Quebec City, Canada; Division of Oral Health and Society, McGill University, Montreal, Canada; Champlain Hospital, Montreal, Canada

**Keywords:** Palliative care, Family medicine, Decision-making, Health communication, Ethnography, Discourse analysis

## Abstract

**Background:**

Conversations about end-of-life care remain challenging for health care providers. The tendency to delay conversations about care options represents a barrier that impedes the ability of terminally-ill patients to participate in decision-making. Family physicians with a palliative care practice are often responsible for discussing end-of-life care preferences with patients, yet there is a paucity of research directly observing these interactions. In this study, we sought to explore how patients and family physicians initiated decision-making conversations in the context of a community hospital-based palliative care service.

**Methods:**

This qualitative study combined discourse analysis with ethnographic methods. The field research lasted one year, and data were generated through participant observation and audio-recordings of consultations. A total of 101 consultations were observed longitudinally between 18 patients, 6 family physicians and 2 pivot nurses. Data analysis consisted in exploring the different types of discourses initiating decision-making conversations and how these discourses were affected by the organizational context in which they took place.

**Results:**

The organization of care had an impact on decision-making conversations. The timing and origin of referrals to palliative care shaped whether patients were still able to participate in decision-making, and the decisions that remained to be made. The type of decisions to be made also shaped how conversations were initiated. Family physicians introduced decision-making conversations about issues needing immediate attention, such as symptom management, by directly addressing or eliciting patients’ complaints. When decisions involved discussing impending death, decision-making conversations were initiated either indirectly, by prompting the patients to express their understanding of the disease and its progression, or directly, by providing a justification for broaching a difficult topic.

**Conclusions:**

Decision-making conversations and the initiation thereof were framed by the organization of care and the referral process prior to initial encounters. While symptom management was taken for granted as part of health care professionals’ expected role, engaging in decisions regarding preparation for death implicitly remained under patients’ control. This work makes important clinical contributions by exposing the rhetorical function of family physicians’ discourse when introducing palliative care decisions.

## Background

Palliative care consists of a holistic and interdisciplinary approach to care that seeks to improve the quality of life of patients and their families when confronted with a life-threatening illness. It encompasses different types of decisions, such as treatment modalities for advanced cancer patients and symptom management for those suffering from terminal chronic illnesses [1]. Research has documented significant variations in the issues that patients, family members, and health care providers deem to be important toward the end of life [2]. Given the preference-sensitive nature of palliative care decisions, patient involvement in decision-making is being promoted as appropriate in this clinical context [3].

Conversations about end-of-life decisions and the transition toward palliative care remain among the most challenging communication tasks for health care professionals [4–6], as initiating palliative care decisions often entails addressing a patient’s impending death. The tendency to delay decisions about advance directives and to focus on curative treatments are major barriers to involving patients in making decisions about palliative care options [7–10]. Opening up decisions for discussion during clinical encounters is therefore the first step toward involving patients in decision-making. As a result, the ability to initiate timely conversations about end-of-life decisions is considered a fundamental skill for palliative care providers [11]. For these reasons, it is important to empirically explore how conversations about sensitive decisions are introduced by experienced palliative care providers during clinical interactions.

Overall, very few studies have analyzed the conversations in which health care providers and palliative care patients engage in decision-making about care options. Expert opinions have been offered to provide guidelines on discussing palliative care, but they rely on case reports and hypothetical scenarios rather than rigorous empirical data [12, 13]. In a recent systematic review of the research directly observing patient-physician discussions in palliative and end-of-life care [14], the studies concerning palliative care patients were found to mainly provide descriptive statistics. More specifically, they reported frequency counts regarding different aspects of talk, such as the different topics discussed [15], explicit encouragement for patient participation in decision-making [16, 17], and the use of euphemisms to avoid discussing death explicitly [18]. Discourse analysis has been used in palliative care to examine the interactional patterns involved in discussions about prognoses [19], as well as the distribution of discursive space in terms of who talked most and about what topics [20]. To the best of our knowledge, no studies using discourse analysis have gathered directly observed, naturally-occurring conversations about decision-making in a palliative care setting [14]; rather, studies often rely on interviews to explore how patients and health care providers justify their decision-making roles [21, 22]. In sum, no research has yet explored how decision-making interactions are initiated through discourse in clinical practice, nor focused on the work of family physicians, who are often responsible for providing end-of-life care in community settings.

Given the importance of addressing decisions early on to ensure that terminally-ill patients have an opportunity to participate in decision-making about their care, we sought to address this important knowledge gap. The question guiding this study was therefore the following: How do terminally-ill patients and their family physicians initiate decision-making conversations about palliative care options in a hospital-based palliative care team? Throughout our research, we aimed to examine the structure of palliative care conversations, to identify how palliative care providers and patients use different types of arguments and discourses to engage in decision-making, and how these discourses were influenced by the context of care. Ethical approval for conducting this study was obtained from the Institutional Review Board of the Faculty of Medicine at McGill University (Certificate A09-E83-09B) and from the ethics committee of the healthcare organization involved in the investigation. Written informed consent for participation in the study was obtained from all participants.

## Methods

### Qualitative methodology

The methodology adopted to answer the research question consists of a combination of discourse analysis with ethnographic observations. A social psychological approach to discourse analysis was selected in order to analyze how decision-making processes are constructed through discourse as well as the social consequences of these processes [23, 24]. For the purpose of this research study, “discourse” is defined as all forms of spoken and non-spoken interactions that construct social reality [23], the social reality in this case being decisions about palliative care options. Ethnographic methods were also borrowed from organizational ethnography, which is a qualitative methodology involving long-term participant observation in an organization in order to understand its culture [25]. During field observations which spanned a year, special attention was paid to the ways in which the context and organization of care influenced the discourse constructing decision-making processes. The methodology aimed to capture the discourse that initiated decision-making conversations during clinical encounters and to examine how this discourse related to the organization of care. This methodological combination represents the best design for obtaining a longitudinal perspective on naturally-occurring discourse during sensitive palliative care interactions as well as for discerning how the organization of care relates to these discourses, taking factors such as long-term doctor-patient relationships into account.

### Study setting

The palliative care providers participating in this study were family physicians and pivot nurses. They were mobile throughout a community acute-care hospital in Montreal, Canada, and also followed patients at the outpatient clinic of the Family Medicine Unit and at home in partnership with a local community health center. The majority of referrals came from the team of oncologists, especially when prognoses worsened and intractable symptoms appeared. However, this study includes referrals from emergency medicine, intensive care, as well as from other family physicians who practiced in the community and did not provide palliative care. The palliative care service also received many referrals from geriatricians, but these patients had cognitive problems that prevented their participation in decision-making and thus precluded their participation in the study. Referrals also occurred whenever patients and families expressed the desire to end active treatments. In 2009, 192 patients being cared for by the team died at the hospital, while 53 patients died at home and 30 patients died after being transferred to a nearby palliative care unit. Patients were generally followed by the same family physician as outpatients or while receiving care at home, unless they experienced an acute episode that needed stabilization as an inpatient, which would be handled by one of the four family physicians on call at the hospital that week. After such an episode, and depending on their condition, patients would either be asked to come back to the outpatient clinic for follow-ups or would receive home visits. The pivot nurses oversaw the continuum of care. If patients and their families decided that death should occur at home, this was arranged with the support of the local community health centers whose nurses performed daily visits and communicated with the palliative care team. If returning home was no longer possible because of medical or social circumstances and unless death was imminent, patients were generally redirected to other services, which included long-term care, or inpatient palliative care units with dedicated beds for when patients’ life expectancy was predictable and limited. There were, however, waiting lists for such services, and it was common for patients to worsen and to die while still under the care of the team in the acute care facility. According to internal statistics, the team estimated that, every year, an average of 40 patients died under their care in the hospital while waiting for a place in a dedicated palliative care unit.

### Data generation

Data were generated over one year of participant observation in the palliative care team (April 2010-2011), where the field researcher (EB) observed interactions from Monday to Friday between 9 a.m. and 5 p.m. at a minimum. Consultations between family physicians and patients recruited for the study were observed across all care settings: the hospital, the outpatient clinic, and at home. Consultations were audio-recorded with the patients’ permission. The field researcher kept detailed field notes of all observations, including consultations, team meetings, informal conversations before and after consultations, and so forth. She was present at all weekly interdisciplinary meetings of the palliative care team and had a desk in the office of the head of the unit, next to the pivot nurses’ office. In addition, she participated in daily rounds with residents when recruited patients were hospitalized and attended the residents’ lectures. A field journal was kept throughout the fieldwork to facilitate reflection during both data gathering and data analysis.

### Recruitment and purposive sampling

Physicians were responsible for recruiting patients and obtaining permission for the researcher to attend the consultation where official consent forms were signed. Patients were included if they were cognitively able to participate in decisions (according to their treating physicians) and spoke English or French. Recruitment occurred through a purposeful sampling procedure [26] by which we sought to recruit new patients under the care of all the different health care providers. The field researcher followed 10 participants at a time in order to reduce scheduling conflicts between observations of inpatient and at-home consultations, which could occur simultaneously when performed by different care providers. She updated the team regarding the need to recruit new patients at the interdisciplinary meetings. A total of 30 patients verbally agreed to participate in the study. However, seven of these patients either deteriorated too rapidly to sign the consent form, or were discharged before signing the consent form and were not readmitted during the duration of the study. Five additional patients were not retained for analysis because there were insufficient data. A total of 18 patients were followed longitudinally throughout the course of their care, and six family physicians as well as two pivot nurses participated in the study (see Table 1 for patient characteristics, origin of referral, and number of consultations observed). Many different types of decisions were observed, including symptom and medication management (pain, anxiety, depression, shortness of breath, nausea, constipation), place of care, radiotherapy, chemotherapy, surgery, antibiotics, do not resuscitate orders, etc.

**Table 1:** **Descriptive characteristics of participating patients**

**Gender**	
Men	8
Women	10
**Age**	
50-59	2
60-69	6
70-79	7
80-89	3
**Diagnosis**	
Cancer	18
Co-morbidity (COPD, heart disease, etc.)	7
**Origin of referral**	
Oncology	8
Family physician	4
Local health service center	3
Emergency care	2
Intensive care	1
**Number of consultations observed**	101
Mean per patient	7
Minimum per patient	1
Maximum per patient	14

### Data analysis

The field notes contained detailed ethnographic data and allowed us to develop a thorough understanding of the context of decision-making processes and of the organizational culture of the palliative care team. The audio-recorded data were transcribed and decision-making conversations were extracted from the discourse (Please refer to the Appendix for the Transcription Key). Decision-making conversations were defined broadly to include all interactions about given topics regarding therapeutic action such as initiating, modifying or stopping a medication, procedures or therapy. We then proceeded to identify the discursive practices initiating all the decision-making conversations identified, searching for differences and consistencies as well as for their function and consequences based on the linguistic evidence at hand, such as the interlocutor’s reaction [23, 24]. Close attention was paid to the types of discursive practices that initiated decision-making conversations, namely the recurring types of questions or arguments that were used to introduce new decisions, and to how these discursive practices related to the organization of care and to contextual features such as the type of decision being introduced, the length of follow-up, and so forth. Data coding involved several iterations as well as the use of HyperRESEARCH 3 qualitative research software. Data were analyzed in their original language; French excerpts were translated into English by the first author and verified by a professional translator. The results show the discursive practices that were found systematically and repeatedly in the data.

## Results

Several aspects of the organizational context and of the decisions at hand appeared to shape the discursive practices that initiated decision-making conversations during clinical interactions between family physicians and patients. The results first address how the organization of care affected decision-making conversations, and then explore the discursive practices involved in initiating different types of decisions, namely decisions about symptom management and about the patients’ future.

### The organization of care and its impact on decision-making conversations

The timeliness of referrals to the palliative care service and the origin of these referrals – i.e. whether requested by the patient or by another health care provider – both affected the discursive practices initiating decision-making about palliative care options. The timeliness and origin of referrals had an impact: (1) on whether patients were still conscious and able to participate in decision-making and could thus be recruited into the study; (2) on the decisions that remained to be made; and (3) on patients’ awareness of their terminal status. It was clear that participating family physicians promoted the importance of introducing decisions about palliative care options early on, before patients could no longer participate in such decisions. This was reflected not only in the way they consistently introduced decision-making conversations early on, but also in the way they taught residents about the need to discuss future options of care and the consequences of failing to do so. For example, during a teaching round observed by the field researcher, a physician (MD 2) gave the example of a surgical intervention that was being done to reverse renal failure in an unconscious, hospitalized patient. The attending physician explained to the residents that such an intervention, while preventing imminent death, could also potentially result in further painful complications. Emphasis was put on the fact that had appropriate discussions occurred earlier, the patient would have had the opportunity to refuse this type of intervention. When patients were referred to the palliative care service at a very late stage in the progression of their illness, their ability to participate in such decisions was reduced, and the decisions that remained to be made mainly revolved around terminal symptom management.

In instances where patients requested a follow-up for symptom control or refused active treatments themselves, physicians did not have to justify their presence and decision-making conversations could proceed in a straightforward manner, because patients discussed their death directly. For example, in the context of a consultation in intensive care, a physician said: *“I’m here to see you because I’ve been told that you wish to stop receiving your transfusions. What makes you say that you’ve had enough?”* The patient replied: *“It’s not improving. I receive the transfusions and two days later I am back here. I’m tired just walking from the bed to the armchair. I’m tired talking and lying down in my bed. It’s not going better”.* Requesting that active treatments be stopped suggests an acknowledgement of terminal status, which the physician then verified by asking: *“And the oncologists have told you that there isn’t any miracle treatment for you?”* To which the patient replied: *“No.”* (Field notes, intensive care consultation, MD 3, Patient 3).

When referrals were made by other health care professionals rather than the patients, family physicians needed to explain what palliative care consists of and to justify the need for their services. This called for a very different way of introducing decision-making conversations from the one in the excerpt above. Throughout the data, health care providers aligned the way they described palliative care to patients with the decisions that had to be made. As can be seen in Excerpt 1, when called to the emergency room for a consultation with a patient diagnosed with lung cancer and suffering from difficulty breathing, a participating physician first emphasized their role as family physicians in charge of controlling pain and difficult symptoms, thus avoiding the label of palliative care, which is often associated with end-of-life care. In contrast, when the consultation had been requested to discuss the pertinence of continuing a given oncological treatment, participating family physicians used openings such as the one we heard in a different encounter: *“I would like to know what you understand from your illness and what has been offered to you. And I would like to know how you see the future”.* (Field notes, inpatient consultation, MD 2, Patient 13). The latter introduction is more in line with a desire to discuss and potentially shift the goal of care.

#### Excerpt 1: decision about management of shortness of breath and anxiety

##### Field notes of emergency room consultation, MD 2, patient 9, family members

*Dr: Hi, I am Dr [name omitted]. I am a family physician. I’m the one who helps with pain and symptom control this week. Here we call it palliative care, but elsewhere it’s called differently. So I’m the one who will be taking care of you this week. Do you have difficulty breathing?*

*P: Yes. Yesterday I wasn’t well at all. They gave me antibiotics and it’s as if it unblocked a little.*

*Dr: Do you spit?*

*P: A bit more since it unblocked.*

*Dr: Ok I will have to verify your scans, but with the radiotherapy, there can be post-radiation inflammation. In that case, the medication that you’re taking, the dexamethasone will help you too. Do you have other symptoms, anything else bothering you? Pain?*

*P: No, I don’t have any pain, only my lump here ((touching neck)).*

*[…]*

*Dr: Tell me, are you anxious?*

*P: No. ((family members nod yes)).*

*Dr: You know, sometimes we don’t see very well inside ourselves, even with glasses. We could ask your family?*

*Family: Yes he is anxious.*

*P: I’m scared to gasp for air.*

*Dr: Are you scared of dying?*

*P: Yes and no, I accept my condition, but I’m scared of suffocating ((family members tear up)).*

*Dr: And I am here to help you with that. It’s important to relieve anxiety also because the more we have difficulty breathing, the more it makes us anxious, and the more we are anxious, the more we tend to breathe so it brings a vicious circle. I will prescribe you a good medication against anxiety that you will be able to take regularly. You know we have very good medications for pain, opioids like morphine and hydromorphone that are also very good medications to help with respiratory problems.*

*P: ((shaking head no)).*

*Dr: But I see that this makes you react, why?*

*P: I don’t want to take medications that will make me need to take more and more and that will make me go.*

*Dr: You know we have to work on a lot of myths about opioids. I can assure you that they are not medications that will be given to you to make you go faster. If you don’t want to take any, I respect that and I am ready to go to the end with the dexamethasone and with your pumps, but you need to know that you have muscles around your lungs, and that you work hard to breathe when you are gasping for air. Sometimes we give opioids and it helps preserve the energy, so that there are people who live longer with the medication.*

A long-term doctor-patient relationship in palliative care also affected the discursive practices initiating decision-making. There was continuity in the decisions introduced during consultations between the same patient-physician dyads, with routine decisions being revisited repeatedly over time during patients’ follow-up appointments. For example, our data include two outpatient consultations that occurred a few weeks apart with the same dyad. The patient suffered from lung cancer that had painfully metastasized into the neck area. The medication to manage the pain involved a long-acting fentanyl transdermal patch, smaller short-acting morphine dosages, as well as methadone pills to attempt to resolve the intractable pain. From one consultation to the next, there was a clear change in the way recurrent decision-making regarding complex pain management with methadone was introduced. During the first consultation, it was the home care nurse who introduced the notion of uncontrolled pain, whereas during the second consultation, the patient initiated the interaction by saying: *“Remind me also about the methadone, we can talk about it”.* (audio-recorded, outpatient consultation, Patient 5, MD 3). There was a routine and familiar quality to the way the decision-making process was introduced in the second consultation, whereby the patient appeared to casually add an item for discussion to the agenda. The discursive practices introducing recurring decisions therefore evolved along with the doctor-patient relationship, so that patients became more familiar with the decisions being made and assumed a more assertive role in initiating decisions.

### Initiating decision-making conversations about symptom management

Decisions regarding symptom management were ubiquitous in the data. Decisions about interventions to alleviate symptoms were directly influenced by patients’ feedback in an attempt to maximize comfort. Symptom control concerned patients’ primary complaints, such as pain, nausea, constipation, shortness of breath, and other types of discomfort. These decisions were either introduced by physicians through general opening questions, or by patients who presented complaints. There was a formulaic quality to the initiation of these decision-making conversations, insofar as they followed a traditional history-taking model. Physicians first asked general open-ended questions, and then followed-up with more specific questions. This was the case in Excerpt 1, where the physician asked a question about the breathing difficulties in general and then specifically about any secretions. The discursive practices of both patients and family physicians constructed decisions about symptom management as needing immediate attention. As such, addressing these decisions did not require justifications, and inquiring about any discomfort represented a common and expected role for family physicians.

For family physicians, engaging in decision-making about symptom management also involved ascertaining whether or not certain complaints from patients indeed needed to be addressed. More specifically, in response to patients who brought up different symptoms requiring decision-making, family physicians would first make sure that patients actually wanted the problem fixed, especially if the symptoms in question were not corroborated with non-verbal displays of discomfort (e.g. frowning and restlessness). In Excerpt 2, the physician verified the relative significance of the symptom in relation to the patient’s level of comfort by asking about sleep and whether the stinging pain was bothersome.

#### Excerpt 2: decision about neuropathic pain management for back fracture

##### Audio-recording of home consultation, MD 3, patient 18, family member

*P: And sometimes it’s eh (.) like prickling (.) like needles […]*

*Dr: This the other medications that you have (.) some amitriptyline?*

*P: No (.) […] [review of medication omitted]*

*Dr: Well for the prickling (.) I could give you (.) do you sleep well?*

*P: Yes I sleep (.) he finds ((referring to family member)) that I don’t sleep well (.) I’m happy with the way I sleep (.)*

*Dr: The prickling it’s caused probably by the pinching at the level of the fracture (.) the nerve sometimes will (.) will be compressed a little and sometimes by a movement it’ll be compressed (.) a little like if you hit your elbow (.) it prickles (.)*

*P: Yes (.)*

*Dr: That that (.) it is possible that (.) well giving you amitriptyline at night 10 or 20 it would diminish this this symptom here (.)*

*P: Yeah (.)*

*Dr: Yeah (.) If it bothers you (.)*

*P: It’s another medication?*

*Dr: Yes (3 s)*

*P: [Yeah but now] (.)*

*Dr: [Yes tell me] (.) I’m listening (.)*

*P: But now another medication it’s that I’ll have that and the other (.) I’d rather wait (.)*

*Dr: Yes that’s it (.) my question is does it really bother you this prickling?*

*P: NO no (.)*

*Dr: If it doesn’t bother you we’re ok we stay like this (.) if it bothers you (.) it could diminish a little this feeling (.) Does your skin burn here?*

*P: No it’s tight (.) it’s tight (.)*

*Dr: Well (.) if you felt like trying it we could know if it diminishes that (.) but if it’s bearable with the long-acting morphine and all eh=*

*P: = Yeah (.)*

*Dr: We’re ok (.)*

*P: Yeah (.) yes yes that’s what I say I’d rather wait (.)*

*Dr: You find that you have enough (.)*

This particular patient had refused many types of interventions over the course of the study, including palliative chemotherapy and anti-coagulants. As the excerpt illustrates, patients oftentimes declined medications for symptoms that they had themselves brought up, which rendered the decision-making process moot. This is relevant because patients tended to raise many symptoms as they discussed how they were feeling. It appears from the data that these discursive practices were not necessarily attempts at introducing decisions, but may also have stemmed from patients’ eagerness to provide complete descriptions of their symptoms and from the therapeutic value of venting during a consultation. As explored below, initiating discussions about worsening health and death involved different discursive practices from the ones used to address symptom management.

### Initiating decision-making conversations about patients’ death

Many palliative care decisions, by default, address the timing and cause of death, making them particularly delicate and emotional. During the consultations observed, there were many decisions about therapeutic options that involved comparing outcomes and thus confronting the future. Once the health of patients declined, conversations about the future would invariably underline their limited life expectancy and entail discussing their death. The daily work of the palliative care team thus involved both symptom management and raising decisions such as the preferred setting for end-of-life care.

In addition to their primary role as requests for more information, patients’ questions during consultations also initiated a change of topic, which in turn led to introducing new decisions. For example in Excerpt 3, a patient asking: *“Where can I go when I cannot be here anymore?”* (audio-recorded, home consultation, MD 4, Patient 6) offered a transition into decision-making about place of care in the future. This patient’s question effectively introduced a decision about the future, and the physician proceeded to list the options available in the area. In our analysis, participating family physicians interpreted direct questions from patients as clear indicators that patients were ready to discuss difficult topics about their approaching death or worsening health, as evidenced by their explicit follow-up with information about end-of-life care.

#### Excerpt 3: decision about place of care

##### Audio-recording of home consultation, MD4, patient 6

*P: Where can I go when I cannot be here anymore?*

*Dr: That’s a very good question (.) There is [Palliative Care Unit 1] (.) and there is [Palliative Care Unit 2] (.) You know those two places? [Palliative Care Unit 1] you went there you had a [friend]*

*P: [I loved] it (.)*

*Dr: Well that would work for you (.)*

*P: I loved it (.)*

*Dr: There is the hospital (.) […] If you can’t stay here anymore or if you decide you can’t stay here anymore (.)*

*P: It could happen tonight (.) My husband is 80 (.)*

*Dr: Exactly (.) There is the hospital (.) Going to the emergency room would be the the the worst option because you would have to wait in the emergency room (.) What we can do is (.) in palliative care we would have you admitted there in palliative care (.)*

*P: That is good and from there they took them to [Palliative Care Unit 1] (.)*

*Dr: Or the best option would be to send you straight from here to [Palliative Care Unit 1] (.) That I think would be the best (.)*

*P: Are there doctors there?*

*Dr: Yes there are doctors there (.) Dr [name omitted] (3 s) he was one of my teachers he’s very good (.) And so that would be another option (.)*

*P: That would be recommended to me by many people […]*

*Dr: But that I think would be the best option (.)*

One way that family physicians initiated discussions regarding impending death was to ask indirect questions about patients’ knowledge or understanding of their condition. In Excerpt 4, the physician introduced the decision about radiotherapy to reduce bleeding associated with uterine cancer by inviting the patient to consider what would happen if the bleeding increased. This led the patient to acknowledge that this could be the cause of her death. With the patient aware of the implications of the bleeding without the physician having to explicitly announce the bad news, the discussion then moved on to the potential benefits and modalities of radiotherapy.

#### Excerpt 4: decision about radiotherapy

##### Audio-recording of consultation at home, MD 4, patient 10, family member

*Dr: Now we talked about the bleeding (.) I’m happy that it stopped (.) it’s reassuring (.) now you have to know (.)*

*P: It hasn’t stopped completely but it’s not as bad (.)*

*Dr: The chances are that during the next months it’s going to start again (.)*

*P: Yes (.) It’s going to start again?*

*Dr: And at some point (.) now we give you a pill to stop the bleeding but it’s possible that at some point the bleeding becomes too strong and that we will not be able to stop it (.) What could happen then in your opinion?*

*P: I don’t know (.)*

*Dr: Let’s say someone loses all his blood (.)*

*P: Ah well yes (.) he could die (.) that’s for sure (.) I have three little pills (.)*

*Dr: Yes you have the iron (.) that’s to stop the bleeding too (.) but it’s possible that at some point it becomes too strong (.)*

*P: Yeah that’s what I’ve been thinking too (.)*

*Dr: And then we have other options at that point to treat you (.) We talked on the phone but I wanted your family to hear too so that we can all make a decision together (.)*

*P: Yeah (.)*

*Dr: We could send her in radiotherapy (.) Now that would mean that they need to do a scan and they would have to do a treatment of radiotherapy (.) that that could diminish the bleeding a little (.) I talked to your mother about it (.)*

*P: Yeah (.)*

*Dr: She says she’s not at all interested (.) What we would get is that it would bleed less (.) you would feel less weak (.) I don’t think it would prolong your life expectancy though (.)*

*P: No I know that too (.)*

*Dr: But it will make you more comfortable for the time that you are here (.) because you’re going to bleed less (.) you’re going to be less weak (.) What do you think?*

*P: There’s always the hope that it will stop because today it seems still but it’s not as bad (.)*

*Dr: It’s not as bad (.) it’s going to get worse (.) diminish (.) worse (.) less (.) it will do like [that]*

*P: [that’s] what it’s doing too (.)*

*Dr: That’s it (.) but it’s possible that it worsens that it goes more and more (.) and then that’s when it would be interesting to do radiotherapy (.)*

*P: But would it stop it completely?*

*Dr: No (.) but it would diminish a lot (.)*

*P: Ah it doesn’t give us anything (.)*

*Dr: You are not at all interested?*

*P: No (.)*

*Dr: It could stop completely but I don’t know (.) that we don’t know (.)*

*P: These things (.) me (.)*

*Dr: It’s not that bad (.) it lasts as I was telling you (.) it’s sessions of five to ten minutes (.)*

*P: But it hurts (.)*

*Dr: It doesn’t hurt (.) it’s radiation (.)*

*P: Ah I’d rather not go (.)*

*Dr: Whatever you want (.) it remains available for you (.)*

*P: Ah (.)*

*Dr: Of course you need to be in good enough shape (.) now you are in good enough shape to go but when we’re very very very very tired then it’s not worth it as much (.)*

While it was expected and routine for physicians to directly ask about pain and symptom management during palliative care consultations, our data suggest that family physicians needed to provide a justification for directly initiating decision-making that went beyond immediate symptom management. Introducing serious decisions that involved talking about the death of a patient was always done carefully in our data, with justifications being given for broaching topics that could engender negative emotions. In Excerpt 5, the physician offered an elaborate justification; the poor health of most hospitalized patients was described, thus giving a routine feel to the decision.

#### Excerpt 5: decision about advance directives

##### Field notes of inpatient consultation, MD 2, patient 13, family members

*Dr: Ok, there is also something that I wanted to talk to you about, it may have been touched on before with you, it’s about what you would like as interventions if you ever suffered medical complications. When we are at the hospital, we’re sick, you agree with me?*

*Family members: Yes.*

*Dr: And when we are hospitalised, we are more at risk for certain complications. Some of them are easy enough to treat, a pneumonia, we give you antibiotics, a urinary tract infection, well. But there are some that are more serious, for example if you have a convulsion that could affect your ability to breathe which requires a respirator to maintain life, or if you ever have a cardiac problem for which we have to do shocks and a cardiac massage. I hope that all this will not happen, but I always like it better if we talk about it while your situation is calm and you are able to participate. If ever you become unconscious then you will not be able to make decisions. So my role, is to give you information and to make sure I know what you need to decide. My medical expertise tells me that in people who have cancer, when there is a minor complication and we treat it it’s quite effective. When there is a major complication like a respiratory or cardiac arrest, the chances that you would come out of it in a good condition are weak. Interventions can prolong suffering and have for result that the patient enters a vegetative state. So I do not want to pressure you one way or another, but my medical recommendation would be to treat you for all infections and other minor problems, but not to resuscitate or intubate you if you have a respiratory or cardiac arrest in order to avoid leaving you in a vegetative state.*

*P: Hallelujah.*

*Dr: Hallelujah, agreed.*

The necessity to address advance directives before patients could no longer participate was also emphasized, while expressing the hope that these directives would prove unnecessary. The fact that physicians’ discursive activities included providing justifications for engaging in decisions about death suggest that patients retained autonomy over non-urgent matters and could decide to face their future on their own terms. Unless death was explored as part of immediate symptom management, as was the case in the decision about the management of terminal anxiety in Excerpt 1, justifications were provided for broaching this topic directly. The justifications encountered in the data included: (1) framing the discussion as a routine exercise for patients of a given age or with a given condition; (2) expressing concern about the likelihood of complications in patients with a given condition; or (3) depicting patient participation as a fleeting opportunity that should be seized before it is too late.

## Discussion

This study sought to understand how palliative patients and their family physicians initiate decision-making conversations about palliative care options. The results first indicate that the organization of care delivery and the types of decisions being made influenced the discourse initiating decision-making conversations. The origin of the referral to the palliative care service, its timing in the patients’ illness trajectory and the development of the therapeutic relationship all shaped whether patients were ready and able to discuss their impending death. Moreover, the study results demonstrate that decisions about symptom management were extremely common during palliative care consultations. These decisions were introduced directly, either through patients’ complaints or through physicians asking questions about symptoms in the traditional history-taking format. In contrast, when decisions entailed discussing an impending death, initiating decision-making conversations was accomplished either indirectly by probing the patients’ understanding of the disease process, or directly by providing a justification for broaching a difficult topic.

This investigation makes important contributions to the understanding of decision-making conversations in a palliative care context. First, the nature of the decision to be made colors the way that patient-physician conversations are undertaken. That is, whereas conversations regarding symptom management involved the use of clear and direct language, conversations regarding death involved asking indirect questions that prompted patients’ to express their understanding of their condition. This discursive practice has been suggested in previous palliative care communication guidelines, which typically encourage health care providers to start from the patient’s perspective, to use open-ended questions, to listen carefully, and to explore emotions [4, 12, 13, 27]. However, the need to justify initiating conversations about death in order to make decisions about palliative care options, as evidenced in this investigation, has not been explicitly recognized in existing clinical guidelines. An article by Roter and colleagues had identified a shift in orientation in clinical conversations, setting discussions about advance directives apart from other routine medical business [28]. Tulsky and colleagues also reported that 93% of the 56 internists participating in their study stated why they were discussing advance directives with patients during clinical conversations [15].

Second, the fact that discussing and planning end-of-life care was implicitly constructed as the patient’s prerogative during clinical discourse could explain why there is a tendency to delay decision-making and to wait for patients to bring up concerns about death and dying themselves [29]. These discursive practices imply that patients retained control over whether or not they would engage in discussions and preparation for death. Previous work has concluded that patients initiate most of the discussions about the future [20], and that oncologists use implicit language to avoid discussing death directly [18]. This study thus concurs with the existing evidence and points to the fact that discussions about a patient’s death remain a highly sensitive and personal topic, even within the practice of family physicians who are experienced palliative care providers. In doing so, this study contributes to highlighting the rhetorical function of the discursive practices recommended in existing clinical practice guidelines. Finally, our investigation contributes to the scarce research directly observing decision-making interactions longitudinally, and was the first to specifically explore how decisions are introduced in the context of palliative care provided by family physicians in a community setting [14].

We employed several verification strategies during the research process to ensure the trustworthiness of the investigation [30]. First, the research question was descriptive and especially designed for discourse analysis, thus ensuring methodological coherence. Sampling adequacy is where this study faced the most threats to validity, given the vulnerable study population. Nonetheless, an effort was made to observe the work of all the physicians and nurses within the team and to recruit a diverse sample with respect to diagnoses, illness trajectories, and socioeconomic levels in order to improve the validity and reliability of the results. Some patients labeled as “difficult” by staff members were also recruited to avoid focusing only on highly proactive patients. We also collected and analyzed data concurrently. In fact, data analysis encompassed several iterations and constantly checking transcripts and notes to make sure that the interpretation could be sustained across the corpus of data. The results were sent to participating health care providers for feedback, although this could not be done with the participating patients due to the fact that they were deceased by the end of the study.

Despite its methodological rigor, this study is not without its limitations. First, the field researcher’s presence may have influenced the way health care providers initiated decision-making conversations with terminally-ill patients; however, given that the researcher was present in the field for a full year, it is likely that the researcher’s influence became minimal. Second, the interactions between family physicians and palliative care patients represented the bulk of the observations, so that the conclusions are more applicable to the work of family physicians providing palliative care in a specific type of community setting than to other members of interdisciplinary palliative care teams. Common limitations related to working with a very sick and vulnerable patient population were encountered. Many patients could not be recruited because they had cognitive problems or experienced too much pain and suffering. Also, first consultations in the inpatient setting could not be audio-recorded because official consent forms first had to be signed and pressing matters had to be attended to by physicians; we did however take detailed notes regarding the interactions during these first consultations.

It is also worth noting that the field researcher had been a palliative volunteer for three years prior to undertaking the fieldwork in this study. This experience played a pivotal role in her ability to display sensitivity to the needs of patients and to adapt recruitment and observation strategies to minimize the inconvenience to patients. Ultimately, research with this patient population is only ethically acceptable if conducted respectfully.

## Conclusion

In closing, this study highlights the fact that discursive practices initiating conversations about palliative care decisions are influenced by the organization of care and by the type of decisions being discussed. Early referral to palliative care shaped the decisions that remained to be made as well as patients’ awareness of their prognosis, while a long-term therapeutic relationship encouraged patients to directly initiate decision-making conversations through affirmations and questions. In cases where patients had not requested the palliative care consultation themselves, family physicians had to justify their presence and aligned the description of palliative care with the decisions at hand. They also offered justifications when directly introducing decisions that entailed discussing death. In the organizational context in which this study took place, the need to discuss options before it was too late represented one such acceptable justification. In contrast, addressing and eliciting patients’ complaints was performed as part of family physicians routine role, was taken for granted and required no such justifications. In summary, our work suggests that directly observing the interactions between terminally-ill patients and family physicians with a palliative care practice can help us better understand how to negotiate challenging conversations regarding end-of-life care in a cultural context where death endures as a highly sensitive topic.

## Appendix

Table 2 contains the transcription key for audio-recorded excerpts.

**Table 2:** **Transcription key for audio-recorded excerpts**

[talk]	Overlapping talk
[overlap]	
(.)	
(2s)	One second pause
60-69	Timed pause in seconds
end of line=	No interval between utterances
=start of line	
?	A rising intonation in speech delivery
!	Exclamation in speech delivery
((laugh))	Contextual information and non-speech sounds
CAPITALS	Louder than surrounding talk
Underline	Emphasis
[...]	Talk omitted from the segment
[name omitted]	Name omitted to protect the anonymity of participants

Adapted from (Wood and Kroger [31] p. 193)
